# Efficacy of Aerial Spraying of Mosquito Adulticide in Reducing Incidence of West Nile Virus, California, 2005

**DOI:** 10.3201/eid1405.071347

**Published:** 2008-05

**Authors:** Ryan M. Carney, Stan Husted, Cynthia Jean, Carol Glaser, Vicki Kramer

**Affiliations:** * California Department of Public Health, Richmond, California, USA; † California Department of Public Health, Sacramento, California, USA; 1 Current affiliation: Yale University School of Medicine, New Haven, Connecticut, USA

**Keywords:** Arboviruses, California, geographic information systems, humans, insect vectors, insecticides, mosquito control, mosquitoes, pesticides, West Nile virus, research

## Abstract

Epidemic transmission of West Nile virus (WNV) in Sacramento County, California, in 2005 prompted aerial application of pyrethrin, a mosquito adulticide, over a large urban area. Statistical analyses of geographic information system datasets indicated that adulticiding reduced the number of human WNV cases within 2 treated areas compared with the untreated area of the county. When we adjusted for maximum incubation period of the virus from infection to onset of symptoms, no new cases were reported in either of the treated areas after adulticiding; 18 new cases were reported in the untreated area of Sacramento County during this time. Results indicated that the odds of infection after spraying were ≈6× higher in the untreated area than in treated areas, and that the treatments successfully disrupted the WNV transmission cycle. Our results provide direct evidence that aerial mosquito adulticiding is effective in reducing human illness and potential death from WNV infection.

## Abstract 2

One-sentence summary for table of contents: Aerial spraying reduced incidence of human infection.

*West Nile virus* (WNV; genus *Flavivirus*, family *Flaviviridae*) is transmitted to humans through the bite of an infected female mosquito and can cause clinical manifestations such as acute febrile illness, encephalitis, flaccid paralysis, and death (*1*). In California, WNV was first identified in 2003, during which time the virus was detected in 6 southern counties and 3 infected persons were identified (*2*). The following year, WNV spread northward from southern California to all 58 counties in the state, resulting in 779 human WNV cases and 28 deaths (*3**,**4*). In 2005, 880 human WNV cases and 19 related deaths were identified in California; 3,000 cases were reported nationwide (*5**,**6*). In contrast to 2004, when most of the WNV activity was concentrated in southern California, activity in 2005 occurred primarily in the northern part of the Central Valley of California, where Sacramento County, the epicenter of WNV activity in the United States that year, had more human cases (163) than any other county in the nation (*7*).

In northern California, the principal urban and rural vectors of WNV are *Culex pipiens* and *Cx*. *tarsalis*, respectively (*8**–**10*). To reduce WNV transmission and human exposure to mosquitoes in 2005, the Sacramento-Yolo Mosquito and Vector Control District (SYMVCD) implemented a battery of control practices from their Integrated Pest Management plan (*11*), an ecosystem-based strategy focused on long-term control of mosquito populations (D. Brown, SYMVCD, pers. comm.). Despite the district’s intensified efforts (which began in March 2005) to control larval mosquitoes and to spot-treat for adult mosquitoes by using truck-mounted equipment, by August 2005 the county had reached the epidemic response level designated by the California Mosquito-Borne Virus Surveillance and Response Plan (*12**,**13*). Per the response plan, SYMVCD determined the appropriate response and control measures through the analysis of 8 surveillance factors, which provided a semiquantitative measure of transmission risk (D. Brown, pers. comm.). Rapidly escalating risk for WNV transmission to humans in Sacramento County was indicated by high mosquito abundance and infection prevalence; high numbers of sentinel chicken seroconversions; and record numbers of dead bird reports, equine cases, and human cases, including ≈24 confirmed human infections by early August (*8**,**10**,**14*). Following state guidelines, and in consultation with local public health officials, SYMVCD initiated aerial adulticiding in Sacramento County in August 2005 to rapidly reduce the abundance of infected mosquitoes and decrease the risk for WNV transmission to humans (D. Brown, pers. comm.). Despite a 60-year history of the aerial application of mosquito control products in California (*15*), this was the first instance within the state of aerial adulticiding over a large urban area.

Although published studies on aerial application of adulticides have documented reductions in mosquito abundance and infection prevalence along with concurrent or subsequent decreases in human cases (*16**–**19*), no published study to date has directly assessed the efficacy of such control efforts in reducing incidence of human disease by comparing distribution of clinical cases within treated and untreated areas. The objective of our study was to evaluate the efficacy of adulticide applications for reducing human cases of WNV; we compared the proportion and incidence of cases in the treated and untreated areas of Sacramento County in 2005 before and after aerial treatments. The proportion and incidence of these cases were also compared with those of the rest of California.

## Methods

### Data Collection

Human WNV case data were reported to the California Department of Public Health from the Sacramento County Department of Health and Human Services and other local health departments throughout the state by using a standardized case history form. A total of 177 human infections were reported within Sacramento County in 2005, with onsets of illness ranging from June through October. Of 177 infections, 163 were clinical cases and 14 were asymptomatic infections; the former was confirmed by immunoglobulin (Ig) G and IgM antibody assays of serum or cerebrospinal fluid samples. Of 163 case records, 7 had no date-of-onset information and 4 others had no residential address. Consequently, the Sacramento County human dataset used in this study comprised 152 records that contained spatial and temporal attributes.

Residential addresses were imported into ArcMap 9.1 geographic information systems software (Environmental Systems Research Institute, Inc., Redlands, CA, USA) and geocoded by using the software’s 2005 StreetMap USA Plus AltNames street dataset. All remaining unmatched addresses were geocoded by using Tele Atlas 2006 (Tele Atlas, Lebanon, NH, USA), NAVTEQ 2006 (NAVTEQ, Chicago, IL, USA.), GDT 2005 (Geographic Data Technology, Inc., Lebanon, NH, USA), and TIGER 2006 (US Census Bureau, Washington, DC, USA) datasets. Population size estimates for the study areas defined below were calculated in ArcMap by selecting census blocks that had their center (centroid) in each defined region (Table 1) (*20*). All data were mapped by using the NAD83 USA Contiguous Albers Equal Area Conic coordinate system.

**Table 1:** Number of human cases of infection with West Nile virus by location and temporal classification, California, 2005*

Area†	Total	Pretreatment‡	Posttreatment§	Postincubation¶	Population#
Treated, northern	34	28	6	0	221,828
Treated, southern	21	20	1	0	338,579
Buffer, northern	13	9	4	3	94,399
Buffer, southern	8	5	3	1	50,127
Untreated	76	41	35	18	518,566
Sacramento County	152	103	49	22	1,223,499
California	670	357	313	197	32,648,149

*Only cases with known date of onset of illness and location information (i.e., Sacramento County at the address level and California at the county level) are included in the analysis. †California excluding Sacramento County. ‡Refers to cases with onset of illness up to and including the last date that aerial adulticiding was conducted (ending 22 Aug for the southern treated area and southern buffer zone and 10 Aug for all other areas). §Refers to cases with onset of illness after the last date that aerial adulticiding was conducted (beginning 23 Aug for the southern treated area and southern buffer zone and 11 Aug for all other areas). ¶Refers to cases with onset of illness >14 days after the first date that aerial adulticiding was conducted (beginning 4 Sep for the southern treated area and southern buffer zone and 23 Aug for all other areas). **#**Population data source: UA Census 2000 TIGER/Line data made available in shapefile format through Environmental Systems Research Institute, Inc. (Redlands, CA, USA) (*20*).

### Adulticide Application

**Figure 1:**
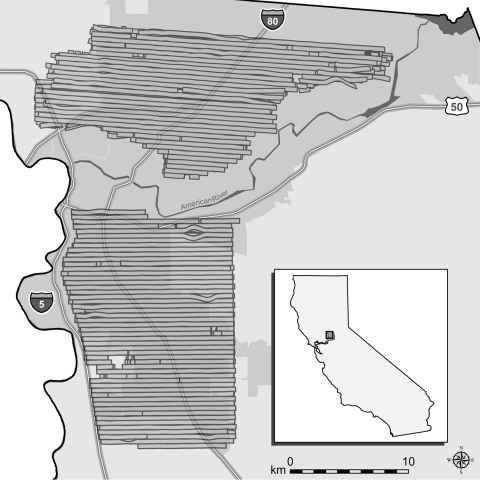
Map of northern and southern aerial adulticiding treatment areas in Sacramento County, California, 2005, showing the 2 urban areas treated by the Sacramento-Yolo Mosquito and Vector Control District (SYMVCD). Horizontal bars represent swaths of spray clouds created by individual passes of the aircraft, as defined by the spray drift modeling systems. Gaps within spray clouds were caused by factors such as towers and buildings that altered the flight of the aircraft (G. Goodman, SYMVCD, pers. comm.). These gaps were assumed to have negligible effect in this study; no human cases occurred within any gaps. Gray region surrounding much of the spray zones represents the urbanized area of Sacramento; urbanized area is defined by the US Census Bureau as a densely settled territory that contains >50,000 persons (*21*). For display purposes, we used the NAD83 HARN California II State Plane coordinate system (Lambert Conformal Conic projection). Inset shows location of treatment areas in California.

Aerial adulticide applications were intended to create aerosolized clouds of insecticide that would contact, and consequently kill, airborne adult *Culex* spp. mosquitoes. SYMVCD targeted areas for treatment on the basis of levels of mosquito infection prevalence that had been previously associated with epidemic transmission within an urban setting (minimum infection rate per 1,000 female *Culex* spp. tested >5.0) (*12*). The district contracted with ADAPCO Vector Control Services (ADAPCO, Inc., Sanford, FL, USA) to apply adulticide by using 2 Piper Aztec aircraft (Piper Aircraft, Inc., Vero Beach, FL, USA) over an area of 222 km^2^ in northern Sacramento County on the nights of August 8–10, 2005 (northern treated area) and an area to the south of 255 km^2^ on the nights of August 20–22, 2005 (southern treated area) (D. Brown, unpub. data) (Figure 1). Coverage was similar each night; repeated applications were intended to increase efficacy (D. Brown, pers. comm.).

The applied compound was Evergreen EC 60–6 insecticide (MGK, Minneapolis, MN, USA), a product composed of 6% pyrethrin/60% piperonyl butoxide (*8*). It was applied at the maximum rate according to the label, 0.0025 pounds of pyrethrins per acre (ultra-low volume dispersal), by 2 Micronair AU4000 atomizer nozzles (Micron Sprayers, Ltd, Bromyard, Herefordshire, UK) on each aircraft, with a swath width of 1,300 feet and expected droplet spectrum volume mean diameters of 32.1 and 36.3 microns for the 2 planes (D. Brown and G. Goodman, unpub. data). Conditions during each night of spraying included wind speeds of 4–10 knots/h and temperatures/dew points of 27°C/14°C (northern treatment) and 33°C/12°C (southern treatment) (D. Brown, unpub. data). Planes began flying at ≈8:00 pm each night and flew for 3–6 h at 130 knots/h (D. Brown, unpub. data). The aircraft flew at altitudes of 61.0 m in the northern treated area and 91.4 m (because of obstacles such as tall towers and buildings) in the southern treated area (R. Laffey, SYMVCD, unpub. data, D. Markowski, pers. comm.). The Wingman GX aerial guidance and recording system (ADAPCO, Inc.), coupled with the Aircraft Integrated Meteorological Management System (AIMMS-20; Aventech Research, Inc., Barrie, Ontario, Canada), modeled the effective drift of released compounds on the basis of real-time meteorologic conditions (D. Brown, pers. comm.). Flight and treatment data were imported into ArcMap for mapping and analysis.

### Case Classification and Analysis

Despite the spray drift modeling systems’ high degree of accuracy, variable and incomplete spray application was expected at the edges of the modeled spray cloud (D. Markowski, pers. comm.). Factors contributing to this phenomenon include the intrinsic margin of error of the aircrafts’ spray drift modeling systems, the extrinsic margin of error caused by factors not detectable or taken into account by the modeling system (i.e., wind gusts, minor changes in aircraft altitude or speed, and other operational variables), and nonoverlapping spray clouds during different nights of application (D. Markowski, pers. comm.). Through consultation with ADAPCO, Inc., this variable and incomplete application at the perimeter was taken into account by delineating a 0.8-km (0.5-mile) buffer within the outermost range of the modeled spray clouds for each treated area (D. Markowski, pers. comm.). Nonbuffered areas of the spray regions (henceforth referred to as treated areas) were considered the most accurate representation of the actual spray application for this analysis, and any WNV cases that occurred within buffer zones were considered separately from those within treated areas. All human cases from Sacramento County that did not occur within treated areas or buffer zones were assigned to the untreated subset of cases, which served as the comparison (control) group for this study.

Cases were further classified by date of onset of illness into pretreatment and posttreatment groups; temporal classification for the untreated area and the rest of California followed that of the northern treated area (Table 1). Because of the relatively lengthy and variable human WNV incubation period, persons who became infected just before the spray events could have become symptomatic up to 14 days later (*22**,**23*). To exclude from analysis any infections that may have been acquired just before the spray events, posttreatment cases that had an onset of illness >14 days after spraying (counting from the first night of application) were also included in a postincubation subset.

The null hypothesis, that the proportion of cases in treated and untreated areas was equal to that of the respective population size estimates, was tested for pretreatment and posttreatment groups with the exact binomial test for goodness of fit by using VassarStats (http://faculty.vassar.edu/lowry/VassarStats.html). Second, significance of proportions of human cases before and after spraying within treated and untreated areas was evaluated with the Fisher exact test of independence by using SAS version 9.1.3 (SAS Institute Inc., Cary, NC, USA). The null hypothesis of this test was that there was no significant association between occurrence of adulticiding and temporal classification of cases (i.e., pretreatment or posttreatment). Third, relative risk (RR) and odds ratio (OR) of infection in the untreated area compared with those in treated areas were calculated by using cumulative incidence of WNV in each region before and after spraying (*24*). To evaluate whether buffer zones had any effect on results, all calculations were repeated by using cases from buffer zones and treated areas combined, as well as cases from buffer zones alone.

### Assumptions

As is standard practice in most epidemiologic studies, residential addresses of patients were assumed to be locations of disease transmission; this is also consistent with other WNV studies (*25**–**31*). The assumption that WNV was transmitted to persons at their place of residence is supported by the fact that WNV mosquito vectors feed primarily from dusk to dawn, and also by findings that persons who spent >2 h outdoors during this time without wearing insect repellant had the highest WNV seroprevalence (*31*).

**Figure 2:**
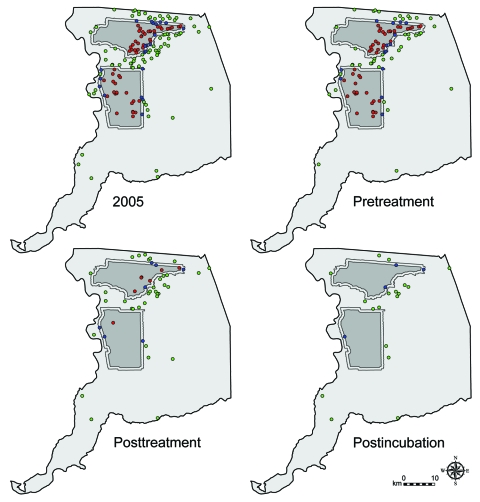
Locations of treated areas and human cases of West Nile virus by temporal classification, Sacramento County, California, 2005. Shown are treated areas (dark gray), surrounding 0.8-km buffers (thin regions around dark gray areas), untreated areas (light gray), and location of human cases within each of these regions (red, blue, and green circles, respectively). For display purposes, we used the NAD83 HARN California II State Plane coordinate system (Lambert Conformal Conic projection).

Because of the random sampling requirement for tests of statistical significance, we must assume that various human populations had an equal likelihood of becoming clinically ill before aerial treatment and that no preexisting factors contributed to a differential in disease experience. Although construction of a multilevel, spatial correlation model is beyond the scope of this study, several important properties of the populations sufficiently support our assumption of homogeneity. Despite the geographic size of the untreated area being ≈6× that of the treated areas combined (2,101 vs. 361 km^2^, Figure 2), population size estimates of both areas were comparable (518,566 vs. 560,407, Table 1) (*20*). Furthermore, the preponderance of cases in the treated (100%, 55/55), buffer (95%, 20/21), and untreated (87%, 66/76) areas was located within the urbanized area of Sacramento, which constitutes 27% (686 of 2,578 km^2^) of the total area of the county (Figure 1) (*20*). Additionally, most cases in the untreated area were located either between the northern and southern treated areas or immediately north of the northern treated area, and >94% (143/152) of all cases were located within 4.8 km (3 miles) of treated areas. This staggered configuration of treated and untreated areas, along with the general proximity of cases within 1 urban region, supported the assumption of homogeneity of populations at risk and created a natural experiment for comparative analyses between treated and untreated areas.

## Results

The observed proportion of pretreatment cases in treated areas to those in the untreated area was not significantly different from the expected proportion on the basis of population size estimates (p = 0.7508, Table 2). Similarly, none of the proportions of pretreatment cases in any combination of treated areas and buffer zones were different from those of the untreated area. However, after adulticiding, all proportions of cases in treated areas were lower than that in the untreated area. Proportions of posttreatment cases in buffer zones were not different from those in the untreated area.

**Table 2:** Statistical test results for West Nile virus cases, Sacramento County, California, 2005*

Area	Goodness of fit†			Independence‡
	Pretreatment	Posttreatment		Posttreatment vs. pretreatment
Treated, both	0.7508	**<0.0001**		**<0.0001**
Treated, northern	0.0650	**0.0391**		**0.0053**
Treated, southern	0.2983	**<0.0001**		**0.0003**
Treated plus buffer, both	0.6195	**<0.0001**		**0.0005**
Treated plus buffer, northern	0.1015	**0.0314**		**0.0069**
Treated plus buffer, southern	0.4568	**<0.0001**		**0.0029**
Buffer, both	0.5140	0.5744		0.3309
Buffer, northern	0.5592	0.5065		0.3745
Buffer, southern	0.5990	1.0000		0.7237

*Numbers of cases were combined for multiple areas; geographically corresponding buffer zones were added where noted. Numbers are 2-tailed p values. Statistically significant associations (p<0.05) are in **boldface**. †Exact binomial goodness-of-fit test for observed proportion of cases in listed area(s) to cases in untreated area compared with the expected proportion based on population size estimates. ‡Fisher exact test of independence for 2 × 2 contingency tables containing numbers of pretreatment and posttreatment cases for listed area(s) and the untreated area.

There was a significantly lower proportion of posttreatment cases within combined treated areas compared with that in the untreated area (p<0.0001, Table 2). Proportions of posttreatment to pretreatment cases within each of the individual treated areas were also significantly lower than that for the untreated area (northern treated area p = 0.0053; southern treated area p = 0.0003). After combining cases from treated areas and buffer zones, proportions of posttreatment versus pretreatment cases were again significantly lower (both treated areas plus buffers p = 0.0005; northern treated area plus buffer p = 0.0069; southern treated area plus buffer p = 0.0029). However, none of the proportions of posttreatment versus pretreatment cases in buffer zones alone compared with those in the untreated area were significantly different (both buffer zones p = 0.3309; northern buffer zone p = 0.3745; southern buffer zone p = 0.7237).

**Figure 3:**
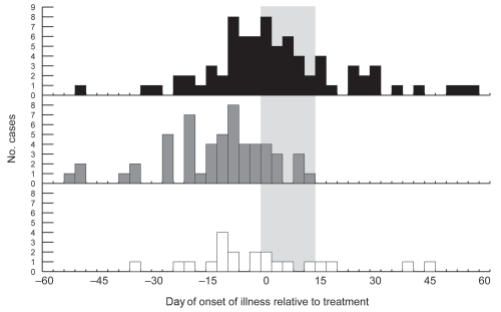
Human cases of West Nile virus (WNV), Sacramento County, California, 2005, by region and date of onset of illness. Black bars show cases within untreated area; gray bars show cases within northern and southern treated areas combined; and white bars show cases within northern and southern buffer zones combined. Values along the x-axis (days) are grouped into sets of 3 and labeled with the date farthest from 0. Each of the 3 days of adulticiding within the treated areas and buffer zones was considered to be 0; for the untreated area, the dates of the northern adulticiding (August 8–10) were considered to be 0. The wide gray vertical band represents time from the first day of treatment to the maximum range of the human WNV incubation period 14 days later.

The last human case that occurred in treated areas had an onset of illness 12 days after inception of spraying, within the 14-day maximum range of the human WNV incubation period. Thus, when the incubation period was taken into account, there were no new human WNV cases reported in either treated area after adulticiding (postincubation cases, Table 1, Figure 3). In contrast, 18 new cases were reported from the untreated area during this time; the last case occurred 59 days after inception of spraying. The frequency of these postincubation cases relative to the overall number of cases in the untreated area (24%) was consistent with that for the rest of the state (29%) but inconsistent with that for treated areas (0%).

**Figure 4:**
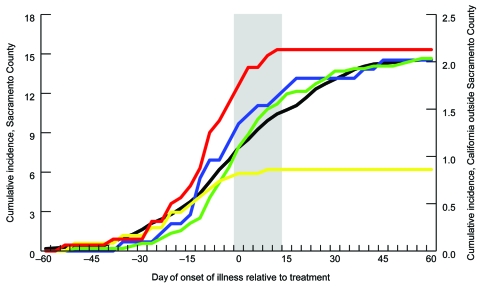
Cumulative incidence of human cases of West Nile virus (WNV) in Sacramento County and California, 2005. Only cases with known date of onset of illness and location information (i.e., Sacramento County at the address level and California at the county level) are included in the analysis. Cumulative incidence is the total no. WNV cases/100,000 population. Green line shows incidence within untreated area; red line shows incidence within northern treated area; yellow line shows incidence within southern treated area; blue line shows incidence within northern and southern buffer zones combined; black line shows incidence within, California excluding Sacramento County. Values along the x-axis (days) are grouped into sets of 3 and labeled with the date farthest from 0. Each of the 3 days of adulticiding within the treated areas and buffer zones was considered to be 0; for the untreated area and the rest of California, the dates of the northern adulticiding (August 8–10) were considered to be 0. The wide gray vertical band represents time from the first day of treatment to the maximum range of the human WNV incubation period 14 days later.

Normalizing number of cases in each region by respective population size estimate showed the increase in incidence levels throughout the year (Figure 4). Statewide (excluding Sacramento County and cases without onset data), cumulative incidence in 2005 was 2.1/100,000 population, and the temporal pattern of incidence throughout the year was similar to that of the untreated area. On the basis of cumulative incidence within each region before aerial treatment, RR for the untreated area compared with that for treated areas was 0.9231 (95% confidence interval [CI] 0.6085–1.400), which did not differ from unity. After treatment, RR was 5.403 (95% CI 2.400–12.16), with an OR of 5.853 (5.403/0.9231, 95% CI 2.351–14.58) in favor of infection in the untreated area than in treated areas; RR and OR differed from unity. Similarly, RRs for the untreated area compared with those for treated areas and buffer zones combined were 0.8990 (95% CI 0.6059–1.334) and 3.398 (95% CI 1.829–6.316) before and after adulticiding, respectively, with an OR of 3.780 (3.398/0.8990, 95% CI 1.813–7.882). Conversely, RRs for the untreated area versus the buffer zones alone were 0.8162 (95% CI 0.4450–1.497) and 1.393 (95% CI 0.6190–3.137) before and after adulticiding, respectively, with an OR of 1.707 (1.393/0.8162, 95% CI 0.6198–4.703); the RRs and OR did not differ from unity.

## Discussion

Evaluation of efficacy is essential for assessing appropriateness of insecticide applications. However, such studies assessing the ability of adulticides to directly affect human incidence of WNV have been nonexistent. Our findings, coupled with corroborating evidence of a reduction in the abundance of *Cx*. *pipiens* (*8*), indicate that aerial application of pyrethrin in 2005 successfully disrupted the WNV transmission cycle, and that this treatment was responsible for an abrupt decrease in the number of human cases within treated areas compared with that in the untreated area. These results provide direct evidence that aerial spraying to control adult mosquitoes effectively reduced human illness and potential deaths from WNV infection.

With respect to population size estimates, proportions of pretreatment cases in all treated areas and buffer zones were not different from that in the untreated area, which validates comparability of the baseline populations. Similarly, none of the pretreatment RRs deviated from unity, which supports the assumption that treated and untreated areas had an equal likelihood, on the basis of population size, of containing a clinical case before the adulticiding, and that no preexisting factors contributed to differing disease incidence rates during that time. These conditions are important for verifying that the untreated area was a valid comparison group for use in statistical analyses.

Comparisons of buffer zones with the untreated area indicated no differences between posttreatment RR or the proportions of posttreatment cases within the 2 areas, which supports the assumption of reduced spray efficacy at the perimeter of the modeled spray cloud. This finding may have implications for future aerial applications and efficacy studies. Additionally, posttreatment infiltration of *Cx*. *tarsalis* mosquitoes from bordering untreated areas has been a previously documented phenomenon in California and Texas (*19**,**32**–**34*). On the basis of mean dispersal distances of *Cx*. *tarsalis* (0.88 km) and *Cx*. *pipiens quinquefasciatus* (1.10 km) in California (*35*), use of the 0.8-km buffer in this study also reduced the probability of including in the treatment groups any human infections contracted through posttreatment mosquito infiltration. However, results of all statistical tests remained unchanged after combining the number of cases from buffer zones and treated areas, and these posttreatment reductions of cases still differed from that in the untreated area (Table 2).

Because posttreatment proportions of cases were lower than in the untreated area, we rejected the null hypothesis of goodness-of-fit comparisons. Our results also indicate that there were associations between adulticiding and temporal classification of cases. Therefore, we also rejected the null hypothesis of tests of independence. Furthermore, odds of infection after spraying were ≈6× higher in the untreated area than in treated areas. Without applications of aerial adulticide, more Sacramento residents would have been infected with WNV. This finding supports federal and California WNV response recommendations, which state that “mosquito adulticiding may be the only practical control technique available in situations where surveillance data indicate that it is necessary to reduce the density of adult mosquito populations quickly to lower the risk of WNV transmission to humans” (*36*).

Although there was a negative correlation between aerial treatments and incidence of human cases, causation is predicated upon spraying having a direct effect on mosquito populations. Recent work showed that adulticiding immediately reduced abundance and infection rates of *Culex* spp. mosquitoes compared with rates in an untreated area (*8*). Using factorial 2-way analysis of variance, these researchers compared mean abundances of *Cx*. *pipiens* and *Cx*. *tarsalis* from CO_2_-baited traps (46 trap nights) in the northern treated area with mean abundances from traps (55 trap nights) in similar urban-suburban habitats within the untreated area of Sacramento County and adjacent Yolo County, 1 week before and 1 week after the August 8 spraying. Abundance of *Cx*. *pipiens* decreased by 75.0%, and there was a significant interaction between adulticiding and temporal classification (*F* 4.965, df 1,47, p = 0.031). Abundance of *Cx*. *tarsalis* decreased by 48.7% but the interaction was not statistically significant (*F* 0.754; df 1,47, p = 0.390). As stated by these researchers, this disparity may have been caused by the presence of “an increasing population of *Cx*. *pipiens* and an already declining population of *Cx*. *tarsalis*” at the time of the spraying, and because *Cx*. *tarsalis* breeds principally in rural areas. Regardless, we reason that *Cx*. *pipiens* was the primary vector in the Sacramento County epidemic because this species is the principal urban vector in this region (*8*–*10*), was the most abundant species collected in Sacramento County in 2005 (D.-E.A Elnaiem, unpub. data), and comprised the highest percentage of WNV-infected mosquito pools (68.3% versus 28.8% for *Cx*. *tarsalis*) in Sacramento County that same year (*10*).

Additionally, these researchers combined mosquitoes of both species (into pools of <50 females) taken from aforementioned traps and others in the northern treated are and untreated area 2 weeks before and 2 weeks after the August 8 adulticiding. Pools of mosquitoes were tested for WNV by using a reverse transcription–PCR, and infection rates were calculated by using a bias-corrected maximum likelihood estimation (www.cdc.gov/ncidod/dvbid/westnile/software.htm). After spraying, infection rates decreased from 8.2 (95% CI 3.1–18.0) to 4.3 (95% CI 0.3–20.3) per 1,000 females in the spray area and increased from 2.0 (95% CI 0.1–9.7) to 8.7 (95% CI 3.3–18.9) per 1,000 females in the untreated area. Furthermore, no additional positive pools were detected in the northern treatment area during the remainder of the year, whereas positive pools were detected in the untreated area until the end of September (D.-E.A Elnaiem, unpub. data). These independent lines of evidence corroborate our conclusion that actions taken by SYMVCD were effective in disrupting the WNV transmission cycle and reducing human illness and potential deaths associated with WNV.

Historically, human WNV cases in the United States peak in August (*37**,**38*). This pattern was observed in Sacramento County and the rest of California in 2005, in which 61% (93/152) and 47% (314/670), respectively, of human cases had onset of illness in August. The next highest month was July, during which 27% (41/152) and 29% (195/670) of human cases had onset of illness in the county and the rest of the state, respectively. These findings are consistent with others from Sacramento County in 2005, which indicated that mosquito infection rates peaked in July and August (*10*). Considering early summer amplification within vector populations and length of the human incubation period, WNV remediation efforts would be more effective in limiting illness and death associated with human infection if conducted at the onset of enzootic amplification rather than after occurrence of human cases.
